# Determining the Organizational Culture and Readiness for Evidence‐Based Practice Amongst Surgical Ward Nurses in Namibia: A Cross‐Sectional Study

**DOI:** 10.1002/hsr2.72825

**Published:** 2026-07-14

**Authors:** Anna Ndapandula Haifete, Petra Brysiewicz

**Affiliations:** ^1^ University of KwaZulu‐Natal KwaZulu‐Natal South Africa

## Abstract

**Background and Aim:**

Evidence‐based practice (EBP) is essential to ensure the delivery of high‐quality healthcare. Integrating evidence‐based practice into the daily work routine of healthcare clinicians has the potential to improve clinical practice. The aim of the study was to determine the organizational culture, readiness, and evidence‐based practice implementation of surgical ward nurses in Namibia.

**Method:**

A hospital‐based cross‐sectional survey was conducted among surgical nurses in two state hospitals in Namibia. Data were collected using two distinct scales. The 19‐item Organizational Culture and Readiness for System‐Wide Integration of Evidence‐Based Practice (OCRSIEP) scale (Melnyk et al., 2010) was used to assess the organizational culture and readiness for EBP. The 18‐item Evidence‐Based Practice Implementation (EBPI) scale (Melnyk et al., 2008) was used to measure the extent of EBP implementation.

**Results:**

Culture and readiness for evidence‐based practice (EBP) were moderate (mean = 81.44, SD = 14.0). Similarly, EBP implementation in daily practice was moderate (mean = 45.83, SD = 17.01). Approximately three‐fifths of participants (*n* = 129; 62.6%) reported opportunities for institutional improvement based on OCRSIEP scores, while more than half (*n* = 113; 54.9%) demonstrated moderate levels of EBP implementation on the EBPI scale. A moderate positive correlation was observed between total OCRSIEP and EBPI scores (*r* = 0.363, *p* < 0.01). Mean OCRSIEP scores differed significantly across departments (*p* = 0.021). Post hoc comparisons indicated statistically significant differences between the state surgical and state gynecological wards (*p* = 0.045), as well as between the state surgical ward and both private and state pediatric wards (*p* = 0.032). *χ* ^2^ analysis indicated no association between gender and OCRSIEP levels (*p* = 0.07).

**Conclusion:**

Moderate levels of organizational readiness and EBP implementation highlight important opportunities for improvement, underscoring the need for targeted strategies to strengthen EBP culture and practice.

## 1 Introduction

The Sustainable Development Goals (SDGs), established by the United Nations in 2015, provide a global framework to achieve equity and sustainability by 2030 [1, 2]. Goal 3: Good Health and Well‐being, emphasizes universal access to safe, high‐quality healthcare, making it directly relevant to nursing, where frontline practice shapes patient safety and outcomes [3]. Evidence‐based practice (EBP) is central to this vision, integrating clinical expertise, patient values, and current research to guide clinical decision‐making [4, 5, 6]. Embedding EBP into nursing practice advances patient well‐being and aligns nursing contributions with global health priorities, thereby positioning nurses as key drivers of SDG3 [7].

Despite global endorsement, EBP implementation (EBPI) remains limited due to barriers such as organizational culture, resource constraints, and insufficient training [8]. This evidence‐practice gap (EPG) reflects the difficulty of translating research into consistent bedside care [9, 10]. Organizational environments strongly influence whether evidence is applied, highlighting the need for supportive conditions [11, 12].

Studies highlighted that organizational culture and readiness are critical determinants of successful EBPI, shaping values, norms, and practices, that prioritize evidence‐based decision‐making [13, 14, 15, 16]. Readiness reflects staff preparedness to adopt new practices [13], influenced by leadership commitment, skilled personnel, and resource availability [17]. Together, culture and readiness underpin EBPI, guiding nurses' attitudes and behaviors [18, 19, 20].

The EBPI is defined as the application of evidence in clinical practice [21] and is recognized as a vital nursing skill [22]. Improving EBP knowledge, skills, and attitudes supports clinicians in adopting high‐quality evidence, particularly when paired with researcher collaboration [23, 24]. However, challenges persist, including limited organizational support, inadequate resources, and insufficient training opportunities [25, 26, 27]. Addressing these barriers requires strategic investment in infrastructure, leadership commitment, and continuous professional development [28]. These challenges extend beyond high‐income countries. In Africa, nations such as South Africa, Ethiopia, Kenya, Nigeria, Egypt, Botswana, Burundi, and Malawi have worked to promote EBP [29], yet, in Ethiopia, EBPI remains particularly constrained by systemic barriers, that hinder the translation of research into routine clinical care [30]. Research capacity in these countries is limited by a shortage of professionals trained in evidence appraisal and implementation science, while financial barriers restrict investment in infrastructure, training, and access to updated scientific resources. Health facilities often lack access to scientific journals, databases, and other digital tools, limiting practitioners' ability to apply evidence [31]. Policy and leadership gaps further weaken institutional support, thereby perpetuating the evidence‐practice gap [30, 31].

In Namibia, EBPI is limited, particularly in surgical wards where postoperative outcomes, infection control, and patient safety depend on rapid, high‐stakes clinical decisions [32, 33], highlighting the need for standardized, evidence‐based protocols [34, 35]. Although, Namibia's Ministry of Health and Social Services launched the National Surgical, Obstetric, and Anaesthesia Plan (NSOAP) 2023/2024‐2026/2027, emphasizing quality assurance and patient safety [36], it does not explicitly address readiness for EBPI.

### 1.1 Study Aim and Objectives

The study aimed to determine organizational culture and readiness for evidence‐based practice (EBP), as well as the extent of EBP implementation, among surgical ward nurses in two state hospitals in Windhoek, Namibia.

The study's specific objectives were to:

- Determine the extent of Organizational Culture and Readiness for System‐Wide Integration of Evidence‐Based Practice (OCRSIEP) and Evidence‐Based Practice Implementation (EBPI) among surgical ward nurses at two state hospitals in Namibia.
- Examine the association between total OCRSIEP and EBPI scores and nurses' demographic characteristics.
- Assess the association between the OCRIESP scores and EBPI scores among surgical ward nurses.
- Determine the association between OCRSIEP levels and nurses' demographic characteristics.

## 2 Research Methods

### 2.1 Study Design

The study employed a quantitative, hospital‐based cross‐sectional study using validated questionnaires namely the OCRSIEP [37] and EBPI [38].

### 2.2 Research Setting

The study took place in Windhoek, Namibia, in the surgical wards of the two state training hospitals that serve as the intermediate referral hospital (Hospital 1) and main national referral hospital (Hospital 2). Hospital 1 and 2, with a bed capacity of 843 and 850, respectively, have various surgical wards/units/departments. Hospital 2 also has a private ward for all disciplines where private patients who underwent surgery are being admitted.

### 2.3 Study Population and Sampling Strategy

The target population was registered nurses working in surgical wards of the two state hospitals (*N* = 206), and a total population sampling method was employed.

#### 2.3.1 Inclusion Criteria

·All registered nurses working in surgical wards during the data collection period, with at least 3 months of experience in the surgical wards.

#### 2.3.2 Exclusion Criteria

- Registered nurses with less than 3 months' experience in surgical wards.

- Registered nurses who were on leave.

### 2.4 Data Collection Instruments

The tool contained sociodemographic characteristics: gender, age, hospital, qualifications, and years/months of experience in the current department. The second section included the OCRSIEP by Melnyk et al. [37] and the EBPI scales developed by Melnyk et al. [38] and revised in 2016. The OCRSIEP Scale is a 19‐item questionnaire using a 5‐point scale (1 = not at all to 5 = very much) to measure the organization's movement towards a culture of EBP. The EBPI is an 18‐item questionnaire using a 5‐point frequency scale to measure how often nurses have performed specific EBP processes in the previous 8 weeks. Responses ranged from 0 = “No activity in the past 8 weeks” to =“Activity performed 8 or more times”. The tool developers granted permission to use these two scales.

### 2.5 Validity and Reliability of the Data Collection Instruments

Organizational culture was measured using the OCRSIEP Scale [37]. Previous studies have established face and content validity for the scale and demonstrated excellent internal consistency reliability above 0.85 across multiple samples [37, 39].

Implementation of EBP was measured with the EBPI Scale [38], which assessed the extent to which the nurses implement EBP. Previous studies have established face and content validity with internal consistency reliability above 0.85 across multiple samples [38, 39]. Cronbach's alpha with this sample was above 0.90. This tool has not been tested in Africa before.

Before the survey, a pilot test of four clinical nurses was conducted to identify and revise any problematic questionnaire items. Adjustment to the piloted tool was made where necessary.

### 2.6 Data Collection Process

To ensure clarity and understanding, a workshop was held on 26 January 2023 to brief participants on the questionnaire before its completion. During the workshop, the researcher explained the aim of the study and assured confidentiality, privacy, and the right to withdraw from the study at any time without prejudice. The researcher obtained written informed consent from the participants at all sites before distributing the questionnaires. The questionnaire took approximately 25–30 min to complete. The researcher created posters with the dates, times (lunch time/tea time), and venue (board rooms/chapel) where the research was conducted. The researcher waited while the participants were completing the questionnaires, and then the completed questionnaires were placed into a strongbox.

### 2.7 Data Analysis

The IBM SPSS version 29 was used for data analysis. Only questionnaires accompanied by a signed informed consent form were considered for analysis. The total OCRSIEP scores were determined by adding the scores of all 19 OCRSIEP statements. The OCRSIEP scale has a total score ranging from 25 to 125. Scores > 75−< 100 demonstrate a moderate movement toward a culture of EBP, but not yet sustainable; scores < 75 indicate an opportunity for growth within the hospital setting to move towards a culture of EBP. A score of ≥ 100 indicated essential movement toward a sustainable culture of organizational readiness and cultural cultivation for implementing EBP.

The total EBPI scores were determined by adding the scores of all 18 EBPI statements. Total scores range from 18 to 90 points, where 0–24 indicates low, 25–48 is moderate, and 49 or higher indicates more frequent practice of EBP processes.

Descriptive statistics included frequencies and percentages. Continuous variables' results are expressed as means and standard deviations. Independent *t*‐tests were performed to ascertain differences in the means of OCRSIEP and EBPI scores based on gender or hospital. Additionally, one‐way ANOVA tests were used to determine if OCRSIEP and EBPI score means varied by age group, years of experience, and qualification. The correlations between OCRSIEP and EBPI scores were determined using Pearson's correlation coefficients. *χ* ^2^ tests were used to assess the associations between the OCRSIEP and EBPI levels and the characteristics of the participants. Binomial logistic regression was performed for variables that had a *χ* ^2^ test *p* value of less than 0.25 to determine the extent of association between OCRSIEP levels and the characteristics of the participants. *χ* ^2^ test *p* values of less than 0.05 and 95% confidence intervals were used to determine the statistical significance of the findings.

### 2.8 Ethical Considerations

Data collection process commenced after ethical approval from the Biomedical Research Ethics Committee (BREC) of the University of KwaZulu‐Natal (BREC/00004372/2022). Permission was granted by the Executive Director of the Ministry of Health and Social Services in Namibia. Following administrative approval from both hospital superintendents, written informed consent was secured from all subjects. The researcher maintained strict participant anonymity and confidentiality, ensuring that involvement remained entirely voluntary.

## 3 Results

### 3.1 Characteristics of Participants

A total of 206 questionnaires were distributed, and a 100% response rate was achieved. The mean age was 32.8 ± 8.06 years, with the majority (*n* = 114; 55.3%) in the age category of 20–30 years, and female (*n* = 172; 83.5%). A statistically significant association existed between qualifications and the hospitals (*p *= 0.004). Table 1 represents demographic differences between hospitals 1 and 2.

**Table 1:** Demographic differences between hospitals 1 and 2.

		Respondents hospital 1 (*N* = 86) *n* (%)	Respondents hospital 2 (*N* = 120) *n* (%)	Test	*p* value
Gender	Male	11 (32%)	23 (68%)	*χ* ^2^ = 1.478	*p* = 0.224
	Female	75 (44%)	97 (56%)		
Age categories	20–30	49 (43%)	65 (57%)	*χ* ^2^ = 3.304	*p* = 0.347
	31–40	25 (41%)	36 (59%)		
	41–50	5 (26%)	14 (74%)		
	> 50	7 (58%)	5 (42%)		
Years/months of experience	Less than 6 months	6 (46%)	7 (54%)	*χ* ^2^ = 8.983	*p* = 0.062
	6–11 months	15 (71%)	6 (29%)		
	1–5 years	38 (37%)	65 (63%)		
	6–10 years	17 (40%)	25 (60%)		
	More than 10 years	10 (37%)	17 (63%)		
Qualification	Diploma	7 (21%)	27 (79%)	*χ* ^2^ = 11.073	***p*** = **0.004**
	Bachelor (Honors) degree	74 (45%)	92 (55%)		
	Master's degree	5 (83%)	1 (17%)		

*Note:* Bold numbers are statistically significant. Chi‐square test (*χ* ^2^); frequency in numbers (*n*); *p* value (*p*).

### 3.2 Organizational Culture and Readiness for System‐Wide Integration of EBP (*N* = 206)

#### 3.2.1 Frequency Distribution of Responses to OCRSIEP Statements

The mean scores of most of the OCRSIEP statements revealed that the organizations had somewhat moved toward organizational culture and readiness for system‐wide integration of EBP (mean scores between 3 and 4). However, four of the statements had mean scores below 3, demonstrating little movement. Table S1 represents the frequency distribution of individual responses to OCRSIEP statements (*N* = 206).

#### 3.2.2 Total OCRSIEP Scores and Frequency Distribution of OCRSIEP Levels of Participants

**Figure 1:**
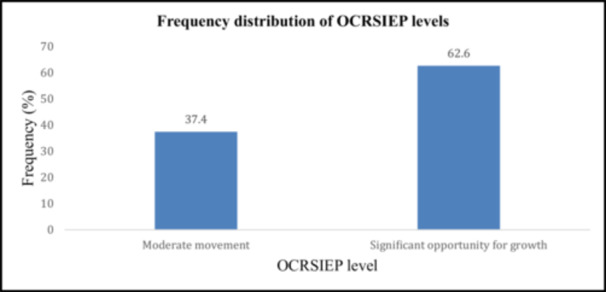
Frequency distribution of OCRSIEP levels of participants.

The mean total OCRSIEP score was 81.44 (SD = 14.0). The lowest score attained by the participants was 36, while the highest score was 111. Almost two‐fifths of the participants (*n* = 77; 37.4%) revealed that their institutions had moderate movement, while about three‐fifths (*n *= 129; 62.6%) reported that their institutions had a significant opportunity for growth. More details on the frequency distribution of OCRSIEP levels are presented in Figure 1.

### 3.3 EBP Implementation

#### 3.3.1 Frequency Distribution of Responses to EBPI Statements

The mean EBPI implementation score was between 2 and 3 for 13 statements, revealing that the participants performed the activities 4–5 times during the past 8 weeks. However, five statements had mean scores of between 2 and 3, indicating that the participants performed the activities 1–3 times during the past 8 weeks. Table S2 represents the frequency of EBP implementation activities “in the past 8 weeks” among surgical ward nurses in Namibia (*N* = 206).

#### 3.3.2 Total EBPI Scores and Frequency Distribution of EBPI Levels of Participants

**Figure 2:**
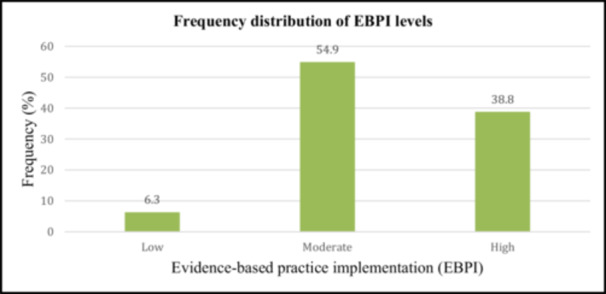
Frequency distribution of EBPI levels of participants.

The mean total EBPI score was 45.83 (SD = 17.01). The lowest score attained by the participants was 19, while the highest score was 84. More than half of the participants (*n* = 113; 54.9%) had moderate EBPI scores, almost two‐fifths (*n* = 80; 38.8%) had high EBPI scores, and less than one‐tenth (*n* = 13; 6.3%) had low EBPI scores. More details on the frequency distribution of EBPI levels are presented in Figure 2.

#### 3.3.3 Correlation Between OCRSIEP and EBPI Scores of Participants

Total OCRSIEP scores have a moderate positive correlation with total EBPI scores (*r* = 0.363, *p* < 0.01).

#### 3.3.4 Association Between the Means of Total OCRSIEP and EBPI Scores and Characteristics of Participants

The means of the total OCRSIEP scores statistically significantly differed by department (*F* [10, 206] = 2.170, *p* = 0.021). However, the means of the total OCRSIEP scores did not differ significantly by gender, age group, years/months of experience, sites, or qualifications (*p* > 0.05). Additionally, the means of the total EBPI scores did not differ significantly by any of the participants' demographic characteristics (*p *> 0.05). More details are in Table S3 representing the association between the means of total OCRSIEP and EBPI scores and characteristics of participants (*N* = 206).

#### 3.3.5 Multiple Comparison of Means of Total OCRSIEP Scores by Department Using Post Hoc Test

Since the ANOVA test among the departments was significant (*p* = 0.021), Tukey Post Hoc Tests were performed further to determine which groups differ. There was a statistically significant difference in total OCRSIEP scores between the state surgery ward and the state gynecological ward (*p* = 0.045), and between the state surgical ward and the private and state pediatric wards for both disciplines (*p* = 0.032).

#### 3.3.6 Association Between OCRSIEP Levels and Characteristics of Participants

The *χ* ^2^ tests showed no statistically significant association between gender and the participants' OCRSIEP levels, *χ* ^2^ (df = 1, *n* = 206) = 3.337, *p* = 0.07. However, no statistically significant association existed between age, years/months of experience, qualification, or hospital and the participants' OCRSIEP levels (*p* > 0.05). Binomial logistic regression did not reveal any association between the participants' characteristics and OCRSIEP levels. More details are in Table 2.

**Table 2:** Association between OCRSIEP levels and characteristics of participants.

Characteristics	Crude odds ratios	95% CI*	*χ* ^2^ test summary		
			Test statistic	Degrees of freedom (df)	*p* value
Age (years)			0.376		*p* = 0.95
20–30	NC	NC			
31–40	NC	NC			
41–50	NC	NC			
> 50	NC	NC			
Gender			3.337	1	*p* = 0.07
Male	2.18	0.93–5.09			
Female	Reference	Reference			
Years/months of experience			2.615	4	*p* = 0.62
Less than 6 months	NC	NC			
6–11 months	NC	NC			
1–5 years	NC	NC			
6–10 years	NC	NC			
More than 10 years	NC	NC			
Qualification			3.378	2	*p* = 0.19
Diploma	0.52	0.25–1.10			
Bachelor's (Honors) degree	Reference	Reference			
Master's degrees	0.52	0.10–2.67			
Hospital			0.695	1	*p* = 0.41
Hospital 1	NC	NC			
Hospital 2	Reference	Reference			

*Note:* Not calculated (NC); *p* value (*p*).

## 4 Discussion

This quantitative study examined the organizational culture and readiness for EBP among surgical ward nurses in Namibia. The overall mean OCRSIEP score of 81.4 indicates a moderate level of organizational readiness for EBP, suggesting that structures and cultural support for EBP are present but not yet fully embedded. In resource‐constrained settings such as Namibia, this level of readiness may reflect ongoing challenges related to access to evidence resources, workforce's capacity, and organizational infrastructure. These findings align with international literature identifying organizational culture, resource limitations, and insufficient training as key barriers to EBP implementation [14, 16, 28, 40], and extend this evidence by demonstrating their persistence within the Namibian surgical ward context. Emerging evidence further highlights the complex interplay between leadership commitment and organizational culture in shaping readiness for EBP [41, 42], suggesting that both structural and relational factors influence the extent to which EBP is prioritized and integrated into daily practice. Collectively, these findings underscore the need for targeted organizational strategies, including leadership engagement and capacity‐building initiatives, to strengthen EBP integration in surgical settings.

High‐scoring OCRIESP items namely, nurse educator champions (item 1), nursing staff commitment to EBP (item 2), physician commitment to EBP (item 3), and belief in organization EBP (item 4) reflected visible leadership support and interprofessional commitment. Conversely, low‐scoring items namely, limited access to databases (item 16), insufficient fiscal resources (item 17), inadequate librarian support (items 19, 20) revealed persistent system‐level barriers, underscoring weak infrastructure and leadership investment, echoing challenges reported in Morocco [11]. These findings highlighted the importance of systemic capacity rather than individual motivation alone in bridging the evidence‐practice gap [43]. Meanwhile, it explains why motivated clinicians in resource‐constrained settings, such as Namibia, struggle to embed EBP [28].

Namibia's policy environment illustrates this disconnection. The Namibian Ministry of Health and Social Services [36] emphasized patient safety and efficiency but omitted explicit strategies for EBPI readiness. This omission is significant, as it reflects a broader policy gap in which quality assurance is pursued without embedding evidence‐based decision‐making at the frontline. By contrast, international benchmarks [43, 44] increasingly integrate readiness assessments into health system strengthening, suggesting that Namibia's approach risks lagging behind global standards. The absence of readiness mechanisms in Namibia's policies, therefore, sustains the evidence‐practice gap, particularly in critical surgical contexts where rapid, evidence‐informed decisions are critical to patient outcomes.

The moderate EBPI score of 45.83 further illustrated the relationship between organizational readiness and individual practice. This aligns with global evidence identifying knowledge gaps, negative attitudes, and low self‐efficacy as barriers to consistent EBPI [8]. Moderate EBPI reflects adequate but inconsistent safety practices, leaving patients vulnerable to preventable harm and falling short of the reliability expected in EBP cultures [45].

The statistically significant positive correlation between OCRIESP and EBPI scores (*r* = 0.363, *p* < 0.01) reinforced the practical significance of organizational culture. Enhancing readiness through leadership commitment, infrastructure, and workforce support may yield measurable improvements in implementation, consistent with the WHO's State of the World's Nursing Report [46]. Departmental differences were also observed, with surgical wards reporting higher scores than gynecological and pediatric wards. These differences may reflect greater exposure to international guidelines [47] and multidisciplinary collaboration [46]. Additionally, gender showed a borderline association with OCRIESP levels (*p* = 0.07), suggesting potential demographic influences that warrant further exploration to determine how these demographic patterns translate into clinical differences.

Together, these findings highlighted persistent structural barriers to EBP in Namibia's surgical wards. Addressing these challenges requires national‐level policy support [46] and targeted workforce development strategies [48] that explicitly embed readiness for EBP. Without such alignment, even well‐intentioned policies risk sustaining the evidence‐practice gap. By situating Namibia's findings within global EBPI debates, this study underscored the need for context‐specific strategies that integrate leadership, culture, and capacity to achieve sustainable improvements in patient outcomes.

### 4.1 Limitations

This cross‐sectional design, reliant on self‐report measures, captured data at a single point in time and is subject to social desirability bias, limiting causal inference. The study scope was restricted to two state hospitals in one region, with a small sample size constrained by nurse shortages, thus reducing generalizability of the findings to similar contexts.

The predominance of younger nurses may have skewed responses, as their exposure to EBP training and institutional support likely differs from that of more experienced colleagues.

### 4.2 Recommendations

This study highlighted the critical role of organizational culture and readiness in supporting EBP among surgical ward nurses in Namibia as follows.

### 4.3 Institutions Recommendations

Investment in infrastructures such as libraries, databases, and reliable internet is critical, alongside dedicated budgets for EBP activities. Digital dashboards and protected time should be introduced to enhance clinical decision‐making. Hospitals should foster a mentorship culture to strengthen support for EBP, as structured mentorship can address gaps in organizational readiness and implementation by providing continuous support, enhancing clinical decision‐making, and promoting the consistent use of evidence in practice.

### 4.4 Policy Recommendations

The Namibian Ministry of Health and Social Services frameworks, and academic initiatives mandating EBP integration are necessary to address systemic gaps. Policymakers should incentivise institutions demonstrating measurable improvements and establishing a centralized EBP resource.

### 4.5 Education Recommendations

Embedding EBP modules across all health science education curricula will better prepare younger nurses. All health science educators' capacity in EBP pedagogy must be strengthened, and clinical‐academic partnerships promoted to bridge theory and practice.

### 4.6 Future Research

Future research should evaluate the scalability and sustainability of organizational culture and readiness for EBP across diverse Namibian healthcare contexts. To enhance generalizability, multi‐site investigations with larger sample sizes are essential. Furthermore, longitudinal studies are needed to assess the long‐term impact of mentorship and digital dashboards on EBP uptake and patient outcomes. Finally, an evaluation of nursing curricula will identify gaps in graduate preparation, providing a basis for evidence‐driven educational reform.

## 5 Conclusion

Organizational culture and readiness are essential for effective EBPI. While nurses' attitudes were positive, moderate levels of culture and implementation scores highlighted the need for stronger institutional support. By identifying gaps in infrastructure, resources, and mentorship, this study contributes to the limited Namibian and African literature on organizational readiness Strengthening supportive environments is essential for translating evidence into routine practice. This is particularly true in surgical wards, where international guidelines and multidisciplinary collaboration serve as models for EBP integration. Ultimately, fostering culture and readiness is key to improving patient outcome.

## Author Contributions

**Anna Ndapandula Haifete:** conceptualization, methodology, investigation, software, data analysis, visualization, writing – original draft. **Petra Brysiewicz:** conceptualization, methodology, data analysis and validation, writing – review and editing, research supervision.

## Funding

The authors have nothing to report.

## Conflicts of Interest

The authors declare no conflicts of interest.

## Transparency Statement

The lead author, Anna Ndapandula Haifete, affirms that this manuscript is an honest, accurate, and transparent account of the study being reported; that no important aspects of the study have been omitted; and that any discrepancies from the study as planned (and, if relevant, registered) have been explained.

## Supporting information


Supporting File 1

Supporting File 2

Supporting File 3

## Data Availability

The data that support the findings of this study are available from the corresponding author upon reasonable request.
