# Supplementary material for: Determining the Organizational Culture and Readiness for Evidence‐Based Practice Amongst Surgical Ward Nurses in Namibia: A Cross‐Sectional Study
Source: Health Sci Rep. 2026 Jul 14;9(7):e72825. doi: 10.1002/hsr2.72825 (PMC13369569; doi:10.1002/hsr2.72825)
Supplement: Supplementary file 1 — Supporting File 1 [file HSR2-9-e72825-s003.docx]

**Supplementary file Table 1: Frequency distribution of individual responses to OCRSIEP statements (N=206)**

| **Item** | **Mean (SD)** | **1**  **n (%)** | **2**  **n (%)** | **3**  **n (%)** | **4**  **n (%)** | **5**  **n (%)** |
| --- | --- | --- | --- | --- | --- | --- |
| 1. Extent to which there is EBP champions in the environment among the Nurse Educators | 3.7 (1.0) | 7 (3.4) | 20 (9.7) | 40 (19.4) | 109 (52.9) | 30 (14.6) |
| 2. Extent to which nursing staff with whom one work with are committed to EBP | 3.7 (0.8) | 1 (0.5) | 12 (5.8) | 63 (30.6) | 104 (50.5) | 26 (12.6) |
| 3. Extent to which the doctors’ team with whom one work with committed to EBP | 3.7 (0.8) | 2 (1.0) | 13 (6.3) | 62 (30.1) | 105 (51.0) | 24 (11.7) |
| 4. Extent to what one believes that EBP is practiced in the organisation | 3.7 (0.7) | 1 (0.5) | 8 (3.9) | 70 (34.0) | 107 (51.9) | 20 (9.9) |
| 5. Extent to which there is a critical mass of nurses who have strong EBP knowledge and skills | 3.6 (0.9) | 5 (2.4) | 17 (8.3) | 70 (34.0) | 84 (40.8) | 30 (14.6) |
| 6. Level of EBP inclusion as central component of the mission and philosophy of the two institutions | 3.6 (0.9) | 2 (1.0) | 18 (8.7) | 66 (32.2) | 90 (43.7) | 30 (14.6) |
| 7. Extent to which there are senior registered nurses who are EBP mentors for other registered nurses | 3.5 (1.0) | 4 (1.9) | 26 (12.6) | 79 (38.3) | 68 (33.0) | 29 (14.1) |
| 8. Extent to which the administrators within the organisation committed to EBP | 3.4 (0.9) | 5 (2.4) | 23 (11.2) | 89 (43.2) | 73 (35.4) | 16 (7.8) |
| 9. Extent to which there is EBP champions in the environment among the doctors | 3.4 (0.9) | 5 (2.4) | 21 (10.2) | 89 (43.2) | 68 (33.0) | 23 (11.2) |
| 10. Extent to which practitioners’ model EBP in their clinical setting | 3.4 (0.9) | 5 (2.4) | 23 (11.2) | 75 (36.4) | 87 (42.2) | 16 (7.8) |
| 11. Extent to which there is EBP champions in the environment among the Senior registered nurses | 3.4 (0.9) | 6 (2.9) | 22 (10.7) | 75 (36.4) | 86 (41.7) | 17 (8.3) |
| 12. Extent to which there is EBP champions in the environment among the Registered nurses | 3.4 (0.9) | 5 (2.4) | 18 (8.7) | 82 (39.8) | 82 (39.8) | 19 (9.2) |
| 13. Extent to which the measurement and sharing of outcomes are part of the culture of the organisation in which one work | 3.3 (0.9) | 10 (4.9) | 21 (10.2) | 87 (42.2) | 73 (35.4) | 15 (7.3) |
| 14. Extent to which registered nurses have proficient computer skills | 3.1 (0.9) | 8 (3.9) | 49 (23.8) | 82 (39.8) | 56 (27.2) | 11 (5.3) |
| 15. Extent to which there is nurse scientists in the organisation to assist in generation of evidence when it does not exist | 3.0 (1.1) | 21 (10.2) | 43 (20.9) | 69 (33.5) | 60 (29.1) | 13 (6.3) |
| 16. Extent to which registered nurses have access to quality computers and access to electronic databases for searching for best evidence | 2.9 (1.1) | 24 (11.7) | 54 (26.2) | 62 (30.1) | 54 (26.2) | 12 (5.8) |
| 17. Extent to which fiscal resources used to support EBP | 2.8 (1.2) | 38 (18.4) | 42 (20.4) | 53 (25.7) | 65 (31.6) | 8 (3.9) |
| 18. Level to which there is EBP champions in the environment among the administrators | 2.7 (1.0) | 20 (9.7) | 73 (35.4) | 67 (32.5) | 35 (17.0) | 10 (4.9) |
| 19. Extent to which librarians within the organisation have EBP knowledge and skills | 2.4 (1.2) | 76 (36.9) | 32 (15.5) | 55 (26.7) | 38 (18.4) | 5 (2.4) |
| 20. Extent to which librarians used to search for evidence | 2.3 (1.2) | 80 (38.8) | 31 (15.0) | 55 (26.7) | 35 (17.0) | 5 (2.4) |
| **Item** | **Mean (SD)** | **None** | **25%** | **50%** | **75%** | **100%** |
| 21. Extent to which decisions are generated from doctors or other healthcare provider groups | 3.5 (0.9) | 6 (2.9) | 15 (7.3) | 84 (40.8) | 82 (39.8) | 19 (9.2) |
| 22. Extent to which decisions are generated from upper administration | 3.4 (0.8) | 4 (1.9) | 17 (8.3) | 100 (48.5) | 68 (33.0) | 17 (8.3) |
| 23. Level to which decisions are generated from direct care providers | 3.2 (0.9) | 7 (3.4) | 33 (16.0) | 87 (42.2) | 67 (32.5) | 12 (5.8) |
| **Item** | **Mean (SD)** | **Not ready** | **Getting ready** | **Been ready but not acting** | **Ready to go** | **Past ready and onto action** |
| 24. Overall rating of the institution in readiness for EBP | 3.4 (0.8) | 3 (1.5) | 23 (11.2) | 79 (38.3) | 92 (44.7) | 9 (4.4) |
| **Item** | **Mean (SD)** | **1**  **n (%)** | **2**  **n (%)** | **3**  **n (%)** | **4**  **n (%)** | **5**  **n (%)** |
| 25. Comparison to 3 months ago, how the movement in the organisation has been toward an EBP culture. | 3.3 (0.8) | 7 (3.4) | 26 (12.6) | 86 (41.7) | 81 (39.3) | 6 (2.9) |

*Standard Deviation (SD); Frequency in numbers (n)*
