# Supplementary material for: Determining the Organizational Culture and Readiness for Evidence‐Based Practice Amongst Surgical Ward Nurses in Namibia: A Cross‐Sectional Study
Source: Health Sci Rep. 2026 Jul 14;9(7):e72825. doi: 10.1002/hsr2.72825 (PMC13369569; doi:10.1002/hsr2.72825)
Supplement: Supplementary file 2 — Supporting File 2 [file HSR2-9-e72825-s001.docx]

**Supplementary file Table 2: Frequency of EBP implementation** **activities “in the past 8 weeks” among surgical ward nurses in Namibia (N=206)**

| Item | Mean (SD) | 0  **n (%)** | 1-3  **n (%)** | 4-5  **n (%)** | 6-8  **n (%)** | >8  **n (%)** |
| --- | --- | --- | --- | --- | --- | --- |
| 1. Has used evidence to change the practice. | 2.3 (1.0) | 9 (4.4) | 32 (15.5) | 77 (37.4) | 70 (34.0) | 18 (8.7) |
| 2. Promoted the use of EBP to colleagues. | 2.2 (1.0) | 13 (6.3) | 32 (15.5) | 88 (42.7) | 51 (24.8) | 22 (10.7) |
| 3.Shared an evidence-based guideline with a colleague | 2.1 (1.3) | 34 (16.5) | 31 (15.0) | 44 (21.4) | 74 (35.9) | 23 (11.2) |
| 4. Changed practice based on patient outcome data. | 2.1 (1.1) | 17 (8.3) | 37 (18.0) | 84 (40.8) | 47 (22.8) | 21 (10.2) |
| 5. Accessed an evidence-based guideline. | 2.0 (1.1) | 29 (14.1) | 37 (18.0) | 62 (30.1) | 72 (35.0) | 6 (2.9) |
| 6. Collected data on a clinical issue. | 1.9 (1.1) | 34 (16.5) | 36 (17.5) | 79 (38.3) | 46 (22.3) | 11 (5.3) |
| 7. Shared the outcome data collected with colleagues. | 1.6 (1.3) | 58 (28.2) | 43 (20.9) | 51 (24.8) | 40 (19.4) | 14 (6.8) |
| 8. Evaluated a care initiative by collecting patient outcome data. | 1.5 (1.2) | 55 (26.7) | 56 (27.2) | 47 (22.8) | 37 (18.0) | 11 (5.3) |
| 9. Shared evidence from a study or studies in the form of a report or presentation to more than 2 colleagues | 1.4 (1.4) | 83 (40.3) | 29 (14.1) | 45 (21.8) | 33 (16.0) | 16 (7.8) |
| 10. Evaluated the outcomes of practice change | 1.4 (1.3) | 74 (35.9) | 40 (19.4) | 38 (18.4) | 39 (18.9) | 15 (7.3) |
| 11. Used an evidence-based guideline or systematic review to change clinical practice where one works. | 1.4 (1.3) | 79 (38.3) | 32 (15.6) | 41 (19.9) | 41 (19.9) | 13 (6.3) |
| 12. Generated a Patient/Population, Intervention, Comparison and Outcomes (PICO) question about practice. | 1.3 (1.4) | 97 (47.1) | 17 (8.3) | 36 (17.5) | 45 (21.8) | 11 (5.3) |
| 13. Critically appraise evidence from a research study. | 1.3 (1.3) | 86 (41.7) | 24 (11.7) | 47 (22.8) | 43 (20.9) | 6 (2.9) |
| 14. Informally discussed evidence from a research study with a colleague. | 1.3 (1.3) | 81 (39.3) | 36 (17.5) | 40 (19.4) | 38 (18.4) | 11  (5.3) |
| 15. Read and critically appraise a clinical research study. | 1.2 (1.3) | 96 (46.6) | 32 (15.5) | 37 (18.0) | 30 (14.6) | 11 (5.3) |
| 16. Shared evidence from a research study with a multidisciplinary team member. | 1.1 (1.4) | 110 (53.4) | 24 (11.7) | 29 (14.1) | 28 (13.6) | 15 (7.3) |
| 17. Shared evidence from a research study with a patient/family member. | 1.1 (1.3) | 108 (52.4) | 25 (12.1) | 34 (16.5) | 27 (13.1) | 12 (5.8) |
| 18. Accessed the Cochrane database of systematic reviews. | 0.9 (1.2) | 115 (55.8) | 30 (14.6) | 33 (16.0) | 24 (11.7) | 4 (1.9) |

*Standard Deviation (SD); Frequency in numbers (n)*
