# Supplementary material for: Determining the Organizational Culture and Readiness for Evidence‐Based Practice Amongst Surgical Ward Nurses in Namibia: A Cross‐Sectional Study
Source: Health Sci Rep. 2026 Jul 14;9(7):e72825. doi: 10.1002/hsr2.72825 (PMC13369569; doi:10.1002/hsr2.72825)
Supplement: Supplementary file 3 — Supporting File 3 [file HSR2-9-e72825-s002.docx]

**Supplementary file Table 3: Association between the means of total OCRSIEP and EBPI scores and characteristics of participants** **(N=206)**

| Variable | Groups compared | N | Mean (SD) | | t/F Value | | p-value | |
| --- | --- | --- | --- | --- | --- | --- | --- | --- |
|  |  | OCRIESP/  EBPI | OCRIESP Score | EBPI Score | OCRIESP | EBPI | OCRIESP | EBPI |
| Sex | Males | 34 | 85.21  (12.10) | 44.97 (17.31) | 1.726 | -0.320 | p=0.192 | p=0.974 |
|  | Females | 172 | 80.69 (14.26) | 45.99 (16.99) |  |  |  |  |
| Age group | 20-30 | 114 | 81.03 (12.98) | 45.36 (16.32) | 0.230 | 1.655 | p=0.876 | p=0.178 |
|  | 31-40 | 61 | 81.95 (13.86) | 45.57 (17.05) |  |  |  |  |
|  | 41-50 | 19 | 83.32 (18.63) | 43.00 (18.29) |  |  |  |  |
|  | >50 | 12 | 79.75 (17.13) | 56.00 (19.78) |  |  |  |  |
| Years/months of experience | Less than 6 months | 13 | 87.85 (14.12) | 47.23 (11.71) | 1.049 | 1.08 | p=0.383 | p=0.368 |
|  | 6 months-11 months | 21 | 82.57 (14.31) | 50.57 (19.05) |  |  |  |  |
|  | 1-5 years | 103 | 81.21 (14.33) | 45.89 (17.04) |  |  |  |  |
|  | 6-10 years | 42 | 79.02 (11.10) | 41.81 (15.47) |  |  |  |  |
|  | >10 years | 27 | 82.07 (16.25) | 47.44 (19.31) |  |  |  |  |
| Sites (Hospitals) | Site 1 | 86 | 80.43 (13.52) | 47.63  (17.45) | -0.873 | 1.29 | p=0.789 | p=0.723 |
|  | Site 2 | 120 | 82.16 (14.34) | 44.53 (16.64) |  |  |  |  |
| Qualifications | Diploma | 34 | 77.35 (18.28) | 45.59 (18.48) | 1.883 | 1.016 | p=0.155 | p=0.364 |
|  | Bachelor (Honours) degree | 166 | 82.35 (12.85) | 46.22 (16.60) |  |  |  |  |
|  | Master’s degree | 6 | 79.33 (15.41) | 36.17 (19.75) |  |  |  |  |
| Department | State surgery ward | 35 | 86.00 (13.80) | 48.66 (16.86) | 2.170 | 1.654 | **p=0.021** | p=0.094 |
|  | State gynaecology ward | 10 | 69.40 (14.48) | 36.90 (9.69) |  |  |  |  |
|  | State orthopaedic ward | 22 | 84.00 (12.72) | 48.55 (18.80) |  |  |  |  |
|  | State urology | 11 | 83.45 (13.00) | 52.45 (17.48) |  |  |  |  |
|  | Private adult ward for both disciplines | 6 | 84.83 (11.27) | 52.50 (18.88 |  |  |  |  |
|  | Private and state paediatric ward for both disciplines | 16 | 71.63 (15.90) | 37.06 (11.68) |  |  |  |  |
|  | Cardiothoracic ward | 3 | 83.67 (10.41) | 27.33 (5.03 |  |  |  |  |
|  | Intensive Care Unit | 24 | 81.46 (13.54) | 43.25 (17.19) |  |  |  |  |
|  | Paediatric ICU ward | 5 | 83.00 (18.66) | 42.20 (19.25) |  |  |  |  |
|  | Surgical and Trauma ICU | 2 | 84.50 (4.95) | 52.00 (21.21) |  |  |  |  |
|  | Theatre | 72 | 81.40 (13.23) | 46.94 (17.22) |  |  |  |  |

*Sample size (N); Standard Deviation (SD); t-test differences between two means (t-value); test differences across multiple means by comparing variances (F-value); p-value (p); Note: Bold numbers are statistically significant*
